# Vascular Contributions to Migraine: Time to Revisit?

**DOI:** 10.3389/fncel.2018.00233

**Published:** 2018-08-03

**Authors:** Bianca N. Mason, Andrew F. Russo

**Affiliations:** ^1^Department of Molecular Physiology and Biophysics, University of Iowa, Iowa City, IA, United States; ^2^Department of Neurology, University of Iowa, Iowa City, IA, United States; ^3^Center for the Prevention and Treatment of Visual Loss, Iowa VA Health Care System, Iowa City, IA, United States

**Keywords:** vascular, migraine, vasodilation, vasoconstriction, inflammation, CGRP

## Abstract

Migraine is one of the most prevalent and disabling neurovascular disorders worldwide. However, despite the increase in awareness and research, the understanding of migraine pathophysiology and treatment options remain limited. For centuries, migraine was considered to be a vascular disorder. In fact, the throbbing, pulsating quality of the headache is thought to be caused by mechanical changes in vessels. Moreover, the most successful migraine treatments act on the vasculature and induction of migraine can be accomplished with vasoactive agents. However, over the past 20 years, the emphasis has shifted to the neural imbalances associated with migraine, and vascular changes have generally been viewed as an epiphenomenon that is neither sufficient nor necessary to induce migraine. With the clinical success of peripherally-acting antibodies that target calcitonin gene-related peptide (CGRP) and its receptor for preventing migraine, this neurocentric view warrants a critical re-evaluation. This review will highlight the likely importance of the vasculature in migraine.

## Introduction

Migraine is a highly prevalent and complex, neurovascular disorder that has been recognized since ancient times. It afflicts approximately 1 in 10 people worldwide and causes a significant socioeconomic burden on society (Smitherman et al., 2013; Burch et al., 2015; Woldeamanuel and Cowan, 2017). Most migraines are diagnosed as episodic, however, approximately 5% of migraineurs experience chronic migraine (>15 headache days monthly; Couch, 2011). As a result, migraine has become a $13 billion dollar industry in direct healthcare costs (Hawkins et al., 2008). The presentation of migraine varies between each individual patient, but a common diagnostic feature is a severe headache with a pulsating quality. The pulsating or throbbing pain underlies migraine being thought of as a vascular disease. The most common treatments, the triptans, are effective in only ~60% of patients and little advancement in effective therapy has occurred since their development (Loder, 2010). However, the promise of monoclonal antibodies for prevention of migraine has reignited the theory of a peripheral input, which most likely involves a vascular contribution. Here, we revisit the vascular basis of migraine.

Theories of migraine pathophysiology have been debated for quite some time. In particular, whether migraine genesis and/or maintenance involves a primary role for the vasculature remains controversial (Goadsby, 2009; Messlinger, 2009; Shevel, 2011). The vascular theory was first articulated by Galen in the second century and later re-proposed by Thomas Willis in the late 17th century (Isler, 1992). However, it wasn’t until the early 1940’s that Harold Wolff first showed that intensity of migraine was closely linked to the pulsating branches of the external carotid arteries and decreasing the amplitude of the pulsations decreased the intensity of the headache (Tfelt-Hansen and Koehler, 2008). In the same study, ergots were used to induce vasoconstriction of temporal and middle meningeal arteries. The vasoconstriction of these vessels reduced throbbing while coincidently diminishing the intensity of the headache. These observations were the genesis of what would later be known as the vascular theory of migraine. However, Ahn reported a study that opposed the observations of Wolff (Ahn, 2010). In a cohort of 20 migraine patients he did not find a temporal relationship between throbbing migraine pain and arterial pulsation.

It seems possible that both intracranial and extracranial arteries play a nociceptive role in migraine (Asghar et al., 2010, 2011; Shevel, 2011). Intracranial vessel dilation has been implicated in migraine, specifically meningeal arteries. In one study, during experimental induction of migraine, dilation of both middle meningeal and middle cerebral arteries was observed (Asghar et al., 2011). During spontaneous migraine, intracranial arteries have been reported to be more dilated during attacks (Amin et al., 2013). For extracranial arteries, the superficial temporal arteries have been reported to be wider on the pain side of the head, and compression of the artery relieved pain in ~30% of patients (Blau and Dexter, 1981; Drummond and Lance, 1983). Additionally, Elkind et al. (1964) concluded that during unilateral headache, blood flow was increased only on the painful side and treatment with ergotamine reduced blood flow and headache in many of the patients. However, it is not clear if ergotamine affected both extracranial and/or intracranial vessels in this study. Other studies used ultrasonography to measure blood flow velocity, a marker for increased artery lumen, during migraine attacks (Thomsen et al., 1995). More recently, studies using magnetic resonance angiography to observe the circumference of extracranial arteries during attacks reached an opposing conclusion that dilation of extracranial arteries was not associated with migraine pain, although there was slight intracranial dilation of the vessels (Amin et al., 2013). Interestingly, both intracranial and extracranial arteries can potentially be innervated by collaterals of the same trigeminal nerve that transverse the skull (Kosaras et al., 2009). Further investigation of both intracranial and extracranial vessels in migraine are necessary.

## Induction of Migraine by Vasoactive Compounds

Using the vascular hypothesis as inspiration, several studies have shown that migraine attacks were associated with release of vasoactive peptides. The vasodilatory peptides calcitonin gene-related peptide (CGRP) and pituitary adenylate cyclase-activating polypeptide (PACAP-38), as well as the neurotransmitter nitric oxide (NO), are all potent vasodilators implicated in migraine pathophysiology (Brain et al., 1985; Moncada et al., 1991; Messlinger et al., 2012; Kaiser and Russo, 2013; Russo, 2015, 2017; Jansen-Olesen and Hougaard Pedersen, 2018).

CGRP is a multifunctional neuropeptide found on sensory nerve fibers (Russell et al., 2014). These fibers innervate vessels where CGRP receptor activation can cause both smooth muscle-dependent and endothelium-dependent activation (Wang et al., 1991; Raddino et al., 1997; Brain and Grant, 2004). Infusion of CGRP in migraine patients results in a delayed migraine-like headache, occurring around 1–5 h after treatment. However, this study also reported a decrease in blood pressure following infusion of CGRP that returned to levels similar to baseline within 60 min of administration (Lassen et al., 2002). To understand the mechanisms of migraine, several studies have induced migraine-like behavior via administration of CGRP (Marquez de Prado et al., 2009; Recober et al., 2009, 2010; Kaiser et al., 2012; Mason et al., 2017; Yao et al., 2017). We have previously reported that overexpression of CGRP receptors in the nervous system enhanced photophobia following central, but not peripheral, administration of CGRP (Mason et al., 2017). These studies suggest that peripheral CGRP had targets outside of nervous tissue that may be relevant in migraine, which is consistent with a possible vascular site of action. However, not all data fit the hypothesis that vasodilation can cause migraine pain. Levy et al. (2005) reported that CGRP-evoked vasodilation failed to induce a nociceptive effect in meningeal nociceptors. Since data focused on vasodilation-induced nociception are conflicting, future studies that dissect whether vasodilation can sensitize dural nociceptors *in vivo* are warranted.

Similar to CGRP, PACAP-38 and NO also can induce migraine. PACAP-38 is present in sensory neurons and on the vascular smooth muscle cells of vessels (Uddman et al., 1993; Mulder et al., 1994; Fahrenkrug and Hannibal, 1998; Tajti et al., 1999; Vaudry et al., 2009). Reports also show that intravenous administration of PACAP-38 can elicit a migraine-like attack in patients (Schytz et al., 2009) and cause sustained meningeal vasodilation and migraine-like photophobia in mice (Markovics et al., 2012). Additionally, both CGRP and PACAP cause vasodilation of the middle meningeal artery (Asghar et al., 2010; Amin et al., 2013; Kaiser and Russo, 2013; Jansen-Olesen and Hougaard Pedersen, 2018). Nitroglycerin (glycerol trinitrate, GTN) is another vasoactive compound implicated in migraine. GTN is enzymatically reduced to NO and/or NO precursors that cause substantial vasodilation (Hill et al., 1992; Millar et al., 1998; Chen et al., 2005; Bonini et al., 2008). GTN administration also causes an immediate headache and a delayed migraine-like headache in migraineurs and a less severe headache in control subjects (Iversen et al., 1989; Thomsen et al., 1994). For these studies, it was reported that during the actual headache phase vasodilation no longer occurs. This is consistent with studies in 1981 by Olesen reporting that the pain was manifested only after vasodilation had subsided (Olesen et al., 1981), although the prolonged initial vasodilatory phase could be important in the pain process. Finally, an important consideration is that vascular agents related to migraine also have neural and/or immune activity (Coleman, 2001; Delgado et al., 2003; Cury et al., 2011; Kaiser and Russo, 2013).

Perhaps the most convincing argument that vasodilation may be an epiphenomenon and not a causative factor in migraine came from a study showing that vasoactive intestinal peptide (VIP) induces vasodilation, but only produced a mild headache, not a migraine (Rahmann et al., 2008). However, the conclusion that vasodilation is an epiphenomenon based on the inability of VIP to induce migraine, may be a premature conclusion. CGRP, PACAP-38 and GTN all cause sustained vasodilation following infusion (Brain et al., 1985; Iversen et al., 1989; Bhatt et al., 2014). In contrast, VIP-induced vasodilation is transitory compared to PACAP-38 (Amin et al., 2014; Edvinsson et al., 2018). Additionally, It was recently reported that PACAP, but not VIP, can dilate the middle meningeal artery (Jansen-Olesen and Hougaard Pedersen, 2018). Hence, there are differences in kinetics and possibly vascular targets of VIP compared to PACAP. Moreover, CGRP, PACAP and GTN cause pro-inflammatory molecule release(Reuter et al., 2001; Raddant and Russo, 2011; Jansen-Olesen et al., 2014; Jansen-Olesen and Hougaard Pedersen, 2018), while VIP is well known for its immuno-protective role and its anti-inflammatory effect (Delgado et al., 2002). Along this line, VIP may also inhibit mast cell degranulation whereas PACAP induces dural mast cell degranulation (Tunçel et al., 2000; Baun et al., 2012). Therefore, it is possible that: (1) increasing VIP infusion time could unveil an ability of VIP to induce migraine-like headaches or (2) the anti-inflammatory properties of VIP play a role in its inability to induce migraine. Future studies with prolonged VIP infusion in migraineurs and examination of cephalic mast cells after infusion of VIP in rodents are warranted.

Although most studies focus on vasodilatory agents implicated in migraine, the role of vasoconstrictors must not be ruled out. There have been observations that plasma levels of endothelin-1 (ET-1), a potent vasoconstrictor, were increased during early stages of a migraine attack, but rapidly decreased at the onset of the headache (Kallela et al., 1998). ET-1 is an important regulator of cerebral blood flow and its receptors are found in endothelium and vascular smooth muscle cells of the arterial system and throughout the CNS (Arai et al., 1990; Sakurai et al., 1990). Kallela et al. (1998) observed that even though ET-1 was elevated during early phases of the migraine attack, the cubital vein blood pressure measurements were unchanged. ET-1 levels rapidly decline approximately 3–4 h after the initiation of the attack, which coincides with reports of headache onset (Kallela et al., 1998). However, the authors did not report the actual blood pressure values and the observation time points are unclear. It is important to note that ET-1 has the ability to either induce vasoconstriction or an initial vasodilation followed by vasoconstriction depending on whether it is activating endothelin type A receptor (ET_A_) or endothelin type B (ET_B_). ET_A_ activation causes sustained vasoconstriction via smooth muscle and can inhibit NO synthesis (Arai et al., 1990; Ikeda et al., 1997) however, ET_B_ activation initially increases release of NO and prostacyclin, which are known vasodilators, followed by sustained vasoconstriction via endothelial cells (de Nucci et al., 1988; Hoffman et al., 1989; Winquist et al., 1989; Sakurai et al., 1990). Furthermore, the non-specific endothelin receptor (A/B) antagonist bosentan inhibits neurogenic inflammation but not vasoconstriction and is not effective for the treatment of migraine (May et al., 1996). However, the ability of bosentan to act on the initial vasodilation that occurs with ET_B_ activation was not assessed. This, along with other studies showing inhibition of neurogenic inflammation is not enough to abort migraine, supports a possible vascular role in migraine. Future studies dissecting the temporal relationship among the release of vasoactive agents such as CGRP, NO and ET-1 in migraine patients are necessary.

## Non-vasodilatory Role of the Vasculature

The vascular aspects of migraine have largely focused on changes in vascular tone, however a non-vasodilatory role of the vasculature has recently been suggested (Jacobs and Dussor, 2016). Vascular inflammation is a mechanism that may contribute to migraine pathogenesis. Dural vessels are thought to contribute to neurogenic inflammation, an event that activates sensory neurons and is characterized by vasodilation, plasma extravasation, and release of pro-inflammatory molecules from mast cells (Raddant and Russo, 2011). These non-vasomotor roles may involve all three layers of vessels: the inner endothelium layer, the middle smooth muscle layer and the outermost adventitia layer of fibroblasts and connective tissue.

The endothelium can both send and respond to signals via release of vasoactive substances to maintain vessel homeostasis (Tomiyama and Yamashina, 2010; Jacobs and Dussor, 2016). For example, when perturbed, cells of the vasculature can release ATP, consequently activate purinergic receptors, stimulate release of NO and pro-inflammatory mediators from endothelial cells (Burnstock, 2016; Jacobs and Dussor, 2016). Endothelium-induced NO release is then capable of sensitizing nearby afferents and possibly contributing to pain experienced during migraine. Two studies reported migraineurs have a decreased count of circulating endothelial progenitor cells (Hill et al., 2003; Rodríguez-Osorio et al., 2012). These cells are a marker of endothelium integrity and function, and a reduction suggest endothelial cell dysfunction (Hill et al., 2003). Additionally, there is mounting evidence of circulating endothelial microparticles in female migraine patients, particularly those diagnosed with migraine with aura (Liman et al., 2015). Tietjen et al. (2009) concluded that decreased concentrations of urinary NO stable metabolites in migraineurs in between migraine attacks compared to control subjects was indicative of endothelial cell dysfunction. Reports from the Levine lab suggest that vascular endothelial cells play a role in enhanced peripheral hyperalgesia via endothelin-1 and both β-adrenergic antagonist ICI-118551 and sumatriptan, both which have receptors on endothelial cells, attenuated endothelin-induced enhancement of hyperalgesia (Joseph et al., 2013). These findings suggest that anti-migraine drugs can produce anti-nociceptive effects by actions on endothelial cells. Conversely, Napoli et al. (2009) concluded that endothelial cells were properly functioning in migraineurs, however, smooth muscle cells failed to function properly following a diminished response to NO. Thus, there is evidence of endothelial dysfunction in migraine, although it is not without controversy.

Smooth muscle cells are of particular interest in migraine although studies that focus on their non-vasomotor contributions are limited. However, one promising area is the ability of NO to activate soluble guanylyl cyclase (sGC) in vascular smooth muscle cells. Recently sGC has been implicated in migraine pathogenesis (Ben Aissa et al., 2017). sGC is a major NO receptor and has been reported as a mediator of nitroglycerin-induced migraine pain (Ben Aissa et al., 2017). While NO induction of sGC causes vasodilation, it can influence dural nociceptors via the NO-cGMP pathway (Levy and Strassman, 2004). More recently Zhang et al. (2013) showed that this NTG infusion has been shown to cause delayed meningeal inflammation via vascular phosphorylated ERK expression. These data, though few, warrant more comprehensive studies to determine the role of smooth muscle activation in migraine models.

Finally, an unexpected contribution of the fibroblasts has recently been suggested by the Dussor lab (Wei et al., 2014). Cultured fibroblasts from the dura are capable of releasing mediators that sensitize dural afferents and induce-migraine-like behavior in rodents (Wei et al., 2014). These cells release IL-6 which is elevated during migraine attacks (Fidan et al., 2006; Sarchielli et al., 2006). It is important to note the fibroblasts used in this study were not cultured from dural vessels and cytokine release was induced by lipopolysaccharide. Future studies that dissect whether adventitial fibroblasts from dural vessels play a role in migraine and a whether a spontaneous mechanism can induce cytokine release are warranted.

## Mast Cell, Neurons and Vessel Cross Talk

The mechanisms that underlie aberrant nociceptor activation are still poorly understood, but are believed to involve changes in the meningeal environment, especially in the dura mater (Zhang et al., 2007, 2010; Zhang and Levy, 2008; Levy, 2009, 2010). The dura mater is a highly vascularized membrane that is heavily innervated by pain fibers and has a dense population of immune cells (Fricke et al., 2001; Jacobs and Dussor, 2016). One theory is that neurogenic inflammation caused by activated mast cells can sensitize nociceptors and thus trigger headache (Theoharides et al., 2005; Waeber and Moskowitz, 2005; Levy, 2009). While direct evidence is lacking, clinical studies have reported increased circulating intracranial inflammatory mediators during an attack (Sarchielli et al., 2006; Goadsby and Edvinsson, 1993). Moreover, activated mast cells release histamine, prostaglandins and a host of pro-inflammatory peptides (Roberts et al., 1979; Heatley et al., 1982; Lewis et al., 1982; Tetlow et al., 1998; Theoharides et al., 2005; Aich et al., 2015). Specifically, tryptase and histamine release have been reported to release neuropeptides from proximal nerve endings and contribute to hyperalgesia (Kleij and Bienenstock, 2005; Aich et al., 2015). This along with the ability of mast cells to increase pERK, cfos, and excitation of meningeal nociceptors provide insight into a mechanism of how mast cells might contribute to peripheral sensitization.

Histamine has been reported to be increased in plasma levels during a migraine attack (Heatley et al., 1982; Moskowitz, 1993; Theoharides et al., 2005). Also, infusion of histamine in migraineurs causes a severe pulsating headache compared to controls (Krabbe and Olesen, 1980). Although with some debate, anti-histamines have been effective in treating migraine in some clinical studies and their potential role in migraine is nicely summarized by Silberstein (Yuan and Silberstein, 2018). Histamine causes dilation of cranial arteries via activation of endothelial histamine receptor H_1_ and inducing formation of NO (Toda, 1990; Ottosson et al., 1991). Moreover, histamine disrupts endothelial barrier formation by altering vascular endothelial cadherin and inducing dilation of vessels (Ashina et al., 2015).

There is an overwhelming abundance of evidence that suggest inflammatory pain states can alter blood brain barrier (BBB) permeability. The BBB is a selective barrier that limits paracellular diffusion via tight junctions between endothelial cells (DosSantos et al., 2014). Given that migraine has increased release of pro-inflammatory peptides, it could involve BBB disruption, although this is still controversial. In a mouse model of cortical spreading depression, the detection of brain edema, plasma extravasation, and altered metalloprotease and matrix proteins were indicative of BBB dysfunction (Gursoy-Ozdemir et al., 2004). In addition to barrier dysfunction, alterations in gap junctions may play a role in migraine. Gap junctions are specialized regions of the plasma membrane that connect cytoplasms of adjacent cells. Tonabersat, a gap junction inhibitor that binds to connexin 43, has been shown to be effective in a subset of migraine patients with aura (Sarrouilhe et al., 2014). Of particular interest, connexin 43 is found on neuronal cells and is one of the connexin proteins associated with cells of the cardiovascular system (Figueroa and Duling, 2009). Given these data, the efficacy of tonabersat suggest a possible role for dysfunction of gap junctions in migraine.

**Figure 1:**
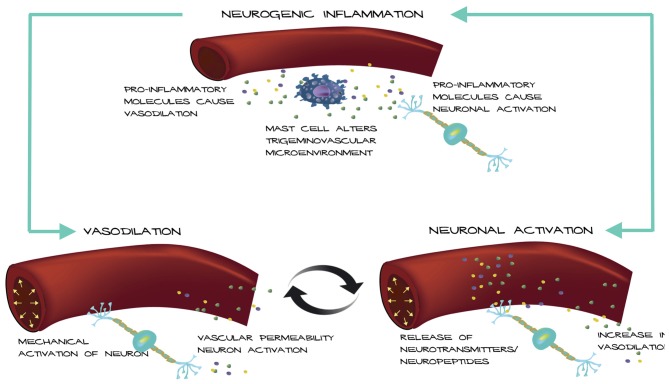
Model of vascular-neural coupling. Mast cell activation and degranulation alter the trigeminovascular microenvironment via release of inflammatory molecules. Inflammatory molecules can mediate vasodilation of nearby vessels and cause nociceptor activation. Vessels can activate trigeminal neurons mechanically or by release of inflammatory mediators due to increased vascular permeability causing a positive feedback loop.

Based on these observations, we propose a possible mechanism for how an altered trigeminovascular microenvironment may initiate vascular-neural cross talk (Figure 1). The meninges are densely vascularized and the layers are innervated by sensory fibers that relay information from the periphery to higher order neurons in the brain. Distention of intracranial blood vessels, possibly from the dura, mechanically activates trigeminal perivascular afferents (Davis and Dostrovsky, 1986; Buzzi et al., 1995). Those activated neurons can release molecules that cause mast cell activation and vasodilation of the nearby vessels in a feed-back loop (Figure 1). In this model, mast cell activation increases vascular permeability and/or causes neuronal activation and neuropeptide release, which causes subsequent release of inflammatory mediators from the vessels that modulate sensory input. Most studies focus on the effect of mast cells on neurons in migraine. Future studies that examine the effect of mast cells on vasculature in translational models of migraine could reveal a role for mast cells in vascular-neural coupling.

## Migraine Treatments and Their Actions on Vessels

Based on the theory that cranial vasodilation was the sole cause of migraine and intravenous infusion of serotonin could successfully treat migraine, a new evidence-based target for migraine therapy was born, the triptans (Humphrey et al., 1989). The triptans have now become the gold-standard in migraine treatment (Ferrari et al., 2001).

Despite the wide use and tolerability of the triptans, the mechanism of action has never been entirely defined. It is known that triptans are 5-HT_1B/D/F_ serotonin receptor agonists that can inhibit the release of neuropeptides involved in migraine and act as vasoconstrictors (Jansen et al., 1992; Nozaki et al., 1992; Williamson et al., 1997; Knight et al., 2001; Wackenfors et al., 2005). Moreover, sumatriptan treatment reduced plasma levels of CGRP in humans (Goadsby and Edvinsson, 1993; Juhasz et al., 2003) and animal models (Buzzi and Moskowitz, 1990; Buzzi et al., 1991; Nozaki et al., 1992). Vascular studies of triptans in people have given insight into its mechanism of action and the roles vessels might play in migraine. In fact, a study using single-photon emission computed tomography combined with Doppler sonography showed that if sumatriptan is infused in people, only the abnormally dilated vessels were reversed back to normal (Asghar et al., 2010, 2011). This observation suggests that triptans only cause significant vasoconstriction on dilated vessels during a migraine. Furthermore, it is interesting to note that triptans work best if used within the first 2 h of the attack (Linde et al., 2006). This coincides with the vasodilatory period following CGRP administration (Lassen et al., 2002). In addition, sumatriptan does not appear to be effective for relieving pain in other disorders (Ahn and Basbaum, 2005), which may be due to the vascular events in migraine. These observations suggest that the vasoconstrictor activity of triptans should not be ignored.

CGRP receptors are found throughout the cranial vasculature. Olcegepant is a non-peptide CGRP antagonist that has high specificity for human CGRP receptors and reported to be efficacious in migraine (Edvinsson, 2008, 2015). Olcegepant blocks dilation of the middle meningeal and extracranial temporal arteries. Similarly, the CGRP receptor antagonist telcagepant has also been shown to inhibit vasodilation of cultured human cerebral and meningeal arteries (Edvinsson et al., 2010). These observations leave open the possibility that some of the CGRP receptor antagonist efficacy might involve the vasculature.

## Association of Migraine With Cardiovascular Disease

Several studies have linked migraine with increased risk of cardiovascular disease. It is reported that migraineurs with aura have a two-fold increased risk for ischemic stroke (Schürks et al., 2009; Sacco and Kurth, 2014). Moreover, patients that experience high frequency migraine have a further increased risk of stroke (Kurth et al., 2009). More to the point, in the largest ever meta-analysis that included over 1 million subjects, migraine has been confirmed to be associated with a higher long-term risk of both ischemic and hemorrhagic stroke and myocardial infarction (Mahmoud et al., 2018). Another study that corroborates a role in migraine is a genome wide association meta-analysis in 2016 by Gormley et al. (2016) that identified 38 susceptibility loci that were enriched for genes associated with arterial tissue. Moreover, several of these genes are associated with smooth muscle dysfunction and cardiovascular disorders linked to migraine as a comorbidity.

There is debate on whether hypertension and/or hypotension have a relationship with migraine (Hagen et al., 2002; Low and Merikangas, 2003; Hamed et al., 2010). It has been suggested that migraine and hypertension have a high prevalence of co-morbidity. Reports that there is a high incidence of migraine in patients with hypertension go as far back as 1913 (Janeway, 1913). Additionally, in a retrospective study, Grebe et al. (2001) found 61% of patients that had developed medicine overuse headache from the treatment of migraine also had hypertension. The same group also found that migraineurs with aura had increased systolic pressure compared to control subjects.

Migraine has a sex disparity and affects a substantial number of women in their reproductive years (Sacco et al., 2012; Finocchi and Strada, 2014). Preeclampsia is a vascular disorder of pregnancy and is characterized by the sudden onset of hypertension and occurrence of vasospasm. It is the leading cause of death among pregnant women (MacKay et al., 2001). In 1959, Rotton et al. (1959) published the first study correlating migraine and preeclampsia and eclampsia in pregnant women. This study noted that a large population of women whose migraine attacks were exacerbated during pregnancy, were also found to have preeclampsia. Moreover, Adeney and Williams used a retrospective approach to examine the association of migraine and preeclampsia and found that 8 out of 10 studies showed a correlation between the two disorders (Adeney and Williams, 2006).

The renin-angiotensin system (RAS), which is involved in hypertension, has been thought to be involved in migraine pathogenesis (Ba’albaki and Rapoport, 2008). Indeed, the efficacy of angiotensin converting enzyme inhibitors in migraine treatment is indicative of a link between migraine and hypertension (Tronvik et al., 2003). Additionally, blood pressure homeostasis is maintained by close communication between the RAS and natriuretic peptides. It has been reported that brain natriuretic peptide (BNP), which is produced by cardiac cells, is elevated in migraine (Uzar et al., 2011). However, BNP is reported to negatively regulate sensory neuron excitability (Vilotti et al., 2013). Further investigations are necessary to elucidate the role of BNP, RAS and hypertension in migraine. Conversely, several studies have also found no correlation between migraine and hypertension (Hagen et al., 2002; Wiehe et al., 2002; Tzourio et al., 2003). In fact, one study suggested that individuals with migraine-like episodes had a higher correlation with lower blood pressure than individuals without headache (Seçil et al., 2010). Furthermore, Seçil et al. (2010) detected diastolic hypotension in normotensive patients at the beginning, during, and up to 1 h following a migraine attack. These observations point to a possible link, but more comprehensive studies are needed to determine if hypertension or hypotension contribute to a sub-population of migraine attacks.

## Conclusion

The ability of vasoactive substances to induce migraine, effective drugs to have a vascular site of action, and the associated correlation of migraine and cardiovascular disease convey that vascular contributions should not be considered an epiphenomenon, but more so a causative component in migraine. Yet, clearly many lines of evidence establish that migraine is a neural disorder. We suggest that the vascular and neural theories can be linked by vascular activation of the nervous system (Figure 1). Understanding the communication between blood vessels, neurons and possibly mast cells will be integral in unraveling the pathophysiology of migraine and future studies should focus on dissecting this intersection of vascular and neural actions in migraine.

## Author Contributions

The manuscript was written by BM and edited by AR.

## Conflict of Interest Statement

AR is a consultant to Alder BioPharmaceuticals, Inc. and has served as a consultant to Eli Lilly and Amgen/Novartis. The remaining author declares that the research was conducted in the absence of any commercial or financial relationships that could be construed as a potential conflict of interest.
